# Double Notched Long-Period Fiber Grating Characterization for CO_2_ Gas Sensing Applications †

**DOI:** 10.3390/s18103206

**Published:** 2018-09-22

**Authors:** Hsiang-Chang Hsu, Tso-Sheng Hsieh, Tzu-Hsuan Huang, Liren Tsai, Chia-Chin Chiang

**Affiliations:** Department of Mechanical Engineering, National Kaohsiung University of Sciences and Technology, NO.415 Jian-gong Road, Kaohsiung, Kaohsiung City 80778, Taiwan; gn1204774@gmail.com (H.-C.H.); srcx2s904@gmail.com (T.-S.H.); iamjohnny825@gmail.com (T.-H.H.); liren@nkust.edu.tw (L.T.)

**Keywords:** sol-gel, CO_2_, gas sensor

## Abstract

In this study, we applied a double-sided inductively coupled plasma (ICP) process to nanostructure long-period fiber grating (LPFG) in order to fabricate a double-notched LPFG (DNLPFG) sensor with a double-sided surface corrugated periodic grating. Using the sol-gel method, we also added thymol blue and ZnO to form a gas sensing layer, thus producing a DNLPFG CO_2_ gas sensor. The resulting sensor is the first double-sided etching sensor used to measure CO_2_. The experimental results showed that as the CO_2_ concentration increased, the transmission loss increased, and that the smaller the fiber diameter, the greater the sensitivity and the greater the change in transmission loss. When the diameter of the fiber was 32 μm (and the period was 570 μm) and the perfusion rate of CO_2_ gas was 15%, the maximum loss variation of up to 3.881 dB was achieved, while the sensitivity was 0.2146 dB/% and the linearity was 0.992. These results demonstrate that the DNLPG CO_2_ gas sensor is highly sensitive.

## 1. Introduction

In recent years, optical fiber has been utilized in an extensive range of applications due to its light weight, immunity to electro-magnetic interference, low power consumption, corrosion resistance, and high temperature resistance [1]. Among the different types of optical fiber, long-period fiber grating (LPFG) has been widely used in the fields of optical communication and sensing systems [2]. LPFG consists of periodic refractive index variations with periods from about 100 μm to 1 mm. The attenuation dip of LPFG is sensitive to environmental parameters and thus can be used to measure and monitor changes in physical parameters. That is, it can be used as an optical fiber sensor.

The processes used to manufacture different types of LPFG include excimer laser writing [3], CO_2_ laser-induced writing [4], the arc discharge technique [5], the mechanical pressure method [6], polyelectrolyte multilayer (PEM) coating [7], and the use of micro-electromechanical systems technology for etching and the application of photoresist [8]. Furthermore, the inductively coupled plasma (ICP) etching technique has been used to fabricate notched LPFG (NLPFG) with a dry etch barrier [9]. In this study, we used the ICP method to fabricate a double NLPFG (DNLPFG) CO_2_ gas sensor. CO_2_ accounts for only 4% of the atmosphere, but the increasing volumes of the CO_2_ in the atmosphere have a major impact on global warming [10]. Therefore, it is important to detect and control the emission of CO_2_ into the atmosphere. In addition, high concentrations of CO_2_ can cause poisoning, and even lead to convulsions, coma, and death [11]. As such, the detection of CO_2_ is a topic very worthy of in-depth study. The existing types of CO_2_ sensors can be divided into semiconductor sensors [12], chemical sensors [13], optical fiber sensors [14], laser diode sensors [15], and non-dispersive infrared (NDIR) sensors [16]. Among those different types, NDIR sensors have been widely employed. However, NDIR sensors have a variety of limitations, such as being bulky and high in cost.

Therefore, some scholars have begun to study the option of coating a sensing layer on an optical fiber to serve as a CO_2_ sensor. For example, Segawa et al. [17] used the sol-gel method to coat thymol blue on optic fiber in order to measure CO_2_ concentrations, finding that the maximum transmission loss variation for their sensor was 2.08 dB, while its sensitivity was about 0.112 dB/%. In contrast, James et al. [18] used pentavinyl hexylamine to sense CO_2_, finding that the maximum transmission loss variation for their sensor was 2.49 dB, while its sensitivity was approximately 0.0727 dB/%. Meanwhile, Wu et al. [19] used NLPFG with an amine-modified surface nanostructure to measure CO_2_, finding that the maximum transmission loss variation for their sensor was 2.019 dB, while its sensitivity was approximately 0.089 dB/%.

In this study, we used the sol-gel method [20] to mix thymol blue with ZnO and then coated that mixture on DNLPFG to create a CO_2_ sensor that is small in size, easy to manufacture, easy to carry, and low in cost.

## 2. Working Principle

The transmission loss of light through LPFG is defined [21] as

(1) $$T=\frac{1}{1+\frac{k^{dc}}{k_{co-cl}^{ac}}}\cos^{2}\left(\sqrt{{\left(k_{co-cl}^{ac}\right)}^{2}+{\left(k^{dc}\right)}^{2}}L\right)$$

where $k^{dc}$, $k_{co-co}^{dc}$, and $k_{cl-cl}^{dc}$ are all LPFG coupling coefficients, $k_{co-cl}^{ac}$ is an AC coupling coefficient, and $k^{dc}$ = $k_{co-co}^{dc}$ = $k_{cl-cl}^{dc}$ =0. Thus, Equation (1) can be simplified to

(2) $$T=\cos^{2}\left(k_{co-cl}^{ac}L\right).$$

The transmission loss of light through LPFG is a cosine squared function, where the transmission loss is dependent on the coupling coefficients and the length of the grating. The combination of CO_2_ gas and sol-gel mix with the thymol blue and nano-ZnO sensing layer changes the effective refractive index for the cladding in Equation (2), thus affecting the coupling coefficient $\left(k_{co-cl}^{ac}\right)$, such that when the gas is detected, the coupling coefficients for the cladding and core become smaller, decreasing the transmission loss. Inputting the experimental data into Equation (2) allows the changes in loss to be determined.

## 3. Manufacturing Process and Experiment Setup

### 3.1. DNLPFG Gas Sensor Manufacturing Process

**Figure 1:**
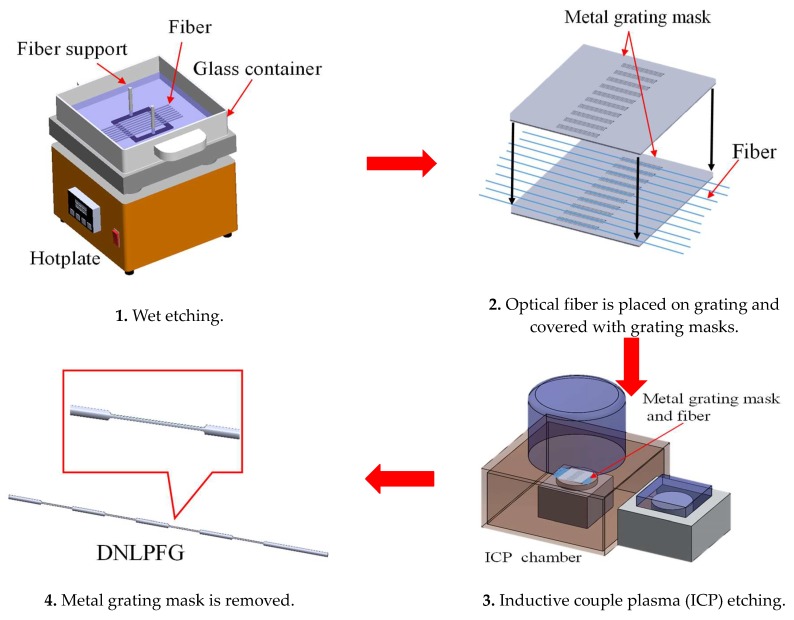
Double-notched long-period fiber grating (DNLPFG) manufacturing process.

**Figure 2:**
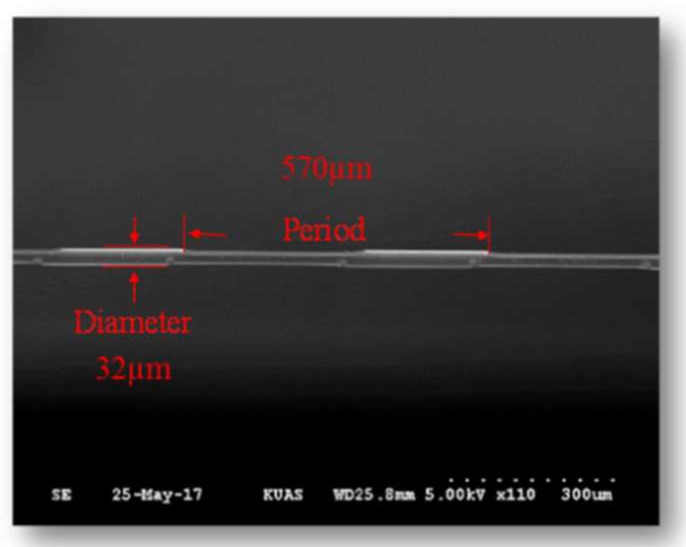
DNLPFG SEM image.

In this study, the double-sided ICP process was applied to single-mode optical fibers (Corning SMF28e) in order to fabricate the DNLPFG sensor with a double-sided surface corrugated periodic grating. This DNLPFG sensor fabrication process is shown in Figure 1. A buffer oxide etch (BOE) chemical was used on the etched fibers to reduce the diameter from 125 to 40, 35, or 32 µm. The fibers were then connected into two metal grating masks (i.e., amplitude masks), and an ICP-etcher was used to generate the double-sided surface corrugated periodic grating of the DNLPFG. Finally, the DNLPFG was obtained after the etched device was released from the metal mask. An SEM image of the DNLPFG sensor is shown in Figure 2.

### 3.2. Sol-Gel Coated DNLPFG CO_2_ Gas Sensor Process

The two different nanoparticles of thymol blue and ZnO were used with a sol-gel to make a gas sensing layer. Etraethoxysilane (TEOS) (4 mL) and *n-octyltriethoxysilane* (Octyl-triEOS) (0.2 mL) were mixed in proportion and then added to and mixed with anhydrous EtOH (1 mL) and hydrochloric acid (HCl) (0.4 mL) to make the sol-gel. The solution was then capped and magnetically stirred for 1 h at room temperature. At this stage, Triton-X-100 (0.1 mL) was added to improve the homogeneity of the silica sol, resulting in a crack-free monolith.

**Figure 3:**
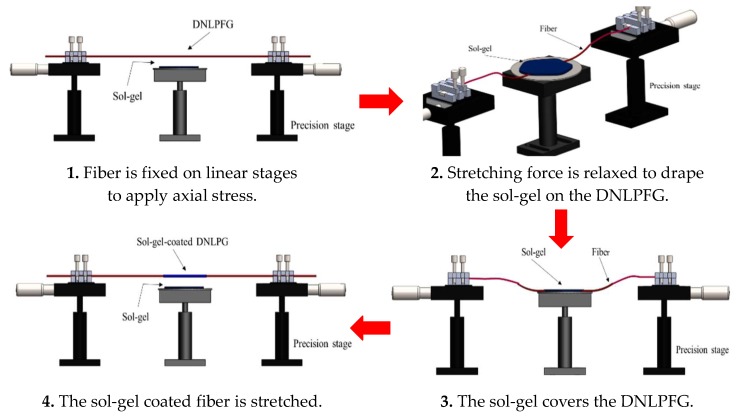
Production flow chart of sol-gel coated DNLPFG sensor.

**Figure 4:**
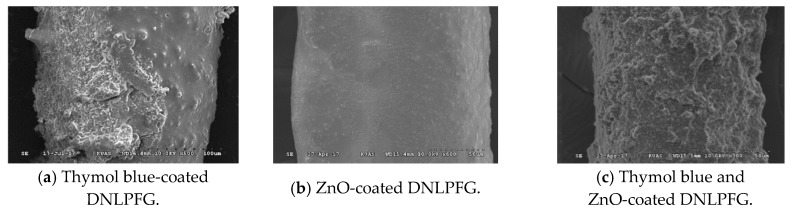
Sol-gel coated DNLPFG CO_2_ gas sensor SEM images.

The optic fiber CO_2_ sensor developed in this study was based on a sol-gel matrix composed of organically modified silica (ORMOSIL) doped with thymol blue, ZnO, or a mix of thymol blue with ZnO nanoparticles to create three different sensing layers, here the ratio of the mixture is 1:1 in volume. The solutions were then draped on the DNLPFG sensor to form a gas sensing layer, as shown in Figure 3. An SEM image of the sol-gel coated DNLPFG sensor is shown in Figure 4.

### 3.3. Packaging of DNLPFG CO_2_ Gas Sensor

**Figure 5:**
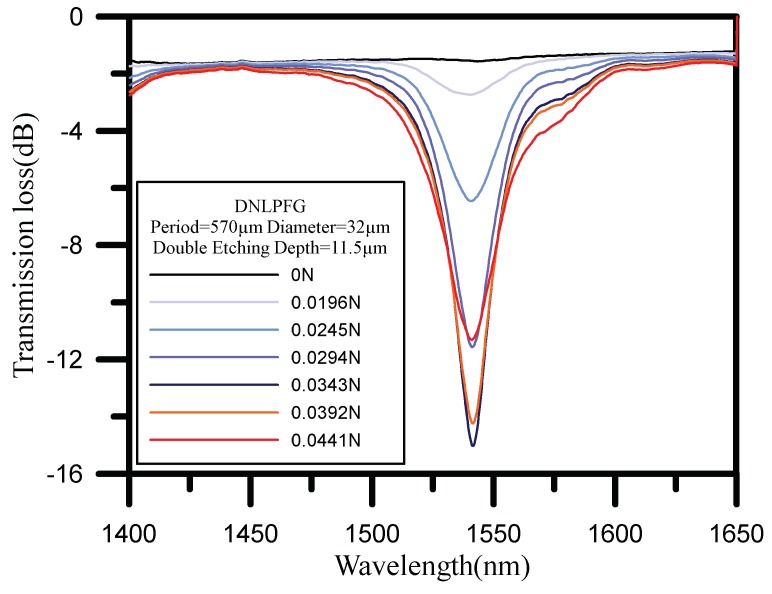
The transmission spectra of DNLPFG sensor under tensile loading.

**Figure 6:**
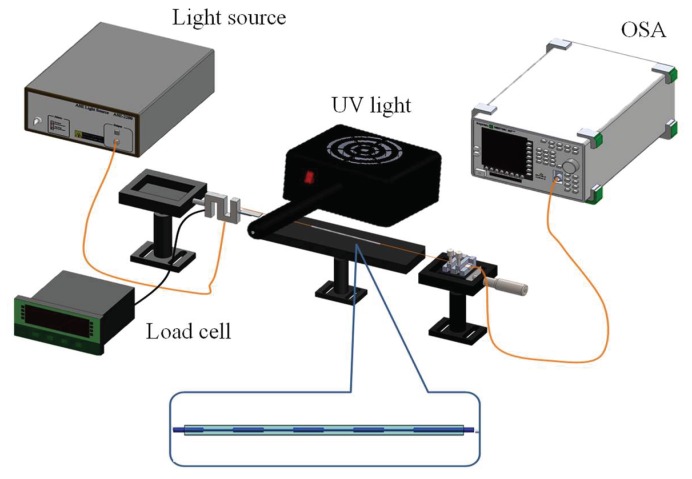
Schematic diagram for packaging of DNLPFG CO_2_ gas sensor.

The spectra of a DNLPFG (with a diameter of 32 µm and period of 570 µm) deform under increased loading, as shown in Figure 5, while the resonant dip of the DNLPFG intensifies with increased loading. When the force loading increases from 0 to 0.0343 N, the resonant dip loss changes from −1.55 to −15.02 dB, and then when the loading increases from 0.0343 to 0.0441 N, this resonant dip loss changes from −15.02 to −11.31 dB. The maximum resonant dip of the DNLPFG sensor was thus −15.02 dB, and the resonant wavelength was 1541.35 nm under 0.0343 N loading. Since the cross section of core of the DNLFBG is small, the applied force to stretch the sensor is small as well. The stretch will make the sensor more sensible. So that, the external axial stress was applied to the sol-gel coated DNLPG gas sensor, it will produce transmission loss for the gas sensing experiments. Because the load element and micro-motion platform to fix the stretching force is inconvenient and difficult to use outdoors, we kept the sensor stretched and fixed on the stage and sleeved with a U-shaped quartz tube, UV glue was then dropped on both ends of the quartz tube. Finally, the UV light was irradiated on the UV glue to package the sensor in a U-shaped quartz tube (shown as Figure 6) The sensor after the package does not required load cell and micro-motion platform, can reduce the experimental equipment, easy to place in the gas experiment chamber.

### 3.4. The Experimental Setup of CO_2_ Gas Sensing

**Figure 7:**
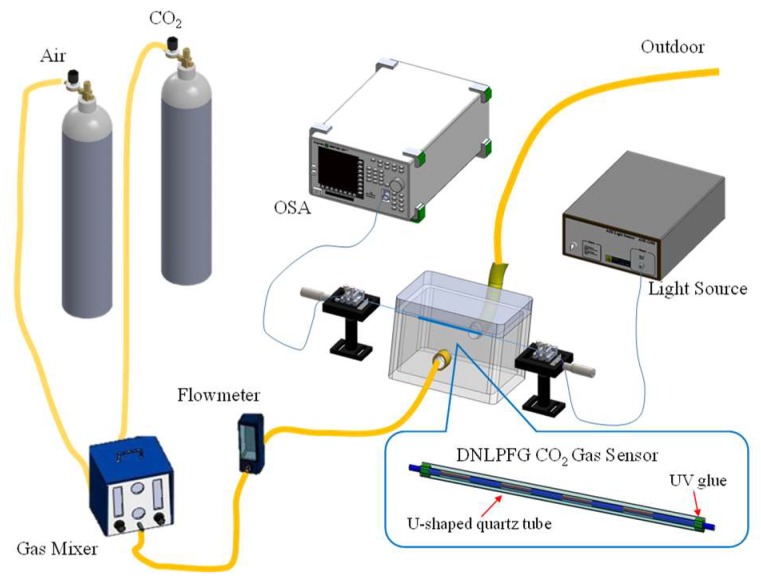
The experimental setup of CO_2_ gas sensing with the sol-gel coated DNLPFG CO_2_ gas sensor.

Figure 7 shows the sol-gel coated DNLPFG CO_2_ gas sensor experimental setup. The sol-gel coated DNLPFG sensor was sealed inside of a U-shaped quartz tube which was in turn placed inside of a gas sensor chamber fitted with a gas loading and unloading system. A gas mixer adjusted the concentration of the CO_2_ gas for experiments using different concentrations of CO_2_. A spectrometer was used to observe the changes in the DNLPFG sensor spectrum after different concentrations of CO_2_ were introduced for analysis.

## 4. Results and Discussion

### 4.1. Response Sensitivity Characteristics of CO_2_ Gas Sensing with Different Sensing Films

A DNLPFG sensor with a diameter of 40 µm and period of 600 µm was used in this experiment. The optic fiber CO_2_ sensor developed in this study was based on a sol-gel matrix composed of ORMOSIL doped with thymol blue, ZnO, or a mix of thymol blue with ZnO nanoparticles to create three different sensing film layers. A spectrometer was used to observe the changes in the DNLPFG sensor spectrum after concentrations of 15% CO_2_ were introduced for 15 min.

**Figure 8:**
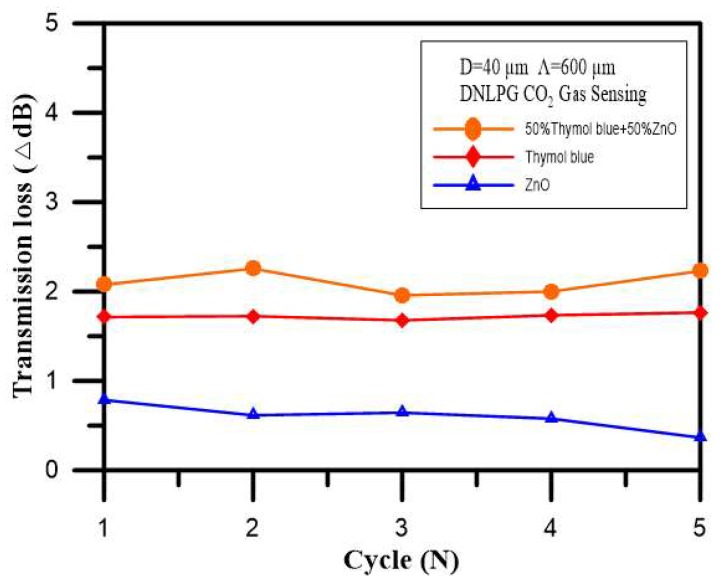
Changes in transmission loss for the CO_2_ gas sensors with different sensing films.

Figure 8 shows the changes in transmission loss for the CO_2_ gas sensor with different sensing films. The figure shows that in the five cyclic experiments, the sensing film layer consisting of a mix of thymol blue with ZnO had the largest transmission loss (1.958–2.257 dB), the sensing film layer consisting of a thymol blue had the second largest transmission loss (1.678–1.765 dB), and the sensing film layer consisting of ZnO had the smallest transmission loss (0.366–0.787dB). The best response sensitivity setting was for the DNLPFG coated with a film of sol-gel matrix composed of ORMOSIL doped with a mix of thymol blue and ZnO.

### 4.2. Response Sensitivity Characteristics of CO_2_ Gas Sensors with Different Diameters

It had been learned from the previous experiment that the DNLPFG coated with the sol-gel mixed with thymol blue solution doped with a mix of thymol blue with ZnO was very effective in terms of CO_2_ gas sensing. The next step was to explore the effects of changes in diameter by measuring CO_2_ using different fiber’s diameters, which would affect the amount of change in transmission loss, sensitivity, and reproducibility. The sensing layer was coated with a sol-gel mixture of thymol blue and zinc oxide. Three DNLPFG sensors with diameters of 40 µm (period of 600 µm), 35 µm (period of 580 µm), and 32 µm (period of 570 µm), respectively, were used in this experiment. The period is calculated referring to the equation in the literature [22].

**Figure 9:**
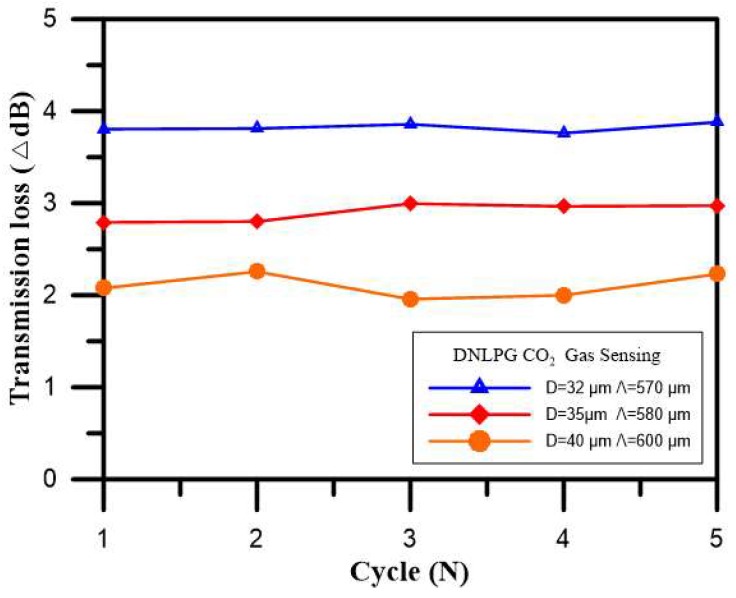
Changes in transmission loss for the CO_2_ gas sensors with different diameters.

Figure 9 shows the changes in transmission loss for these CO_2_ gas sensors with different diameters. The figure shows that in the five cyclic experiments for the three types DNLPFG, the DNLPFG sensor with a diameter of 32 µm had the largest transmission loss (3.762–3.881 dB), the DNLPFG sensor with a diameter of 35 µm had the second largest transmission loss (2.793–2.996 dB), and the DNLPFG sensor with a diameter of 40 µm had the smallest transmission loss (1.958–2.257 dB). The best response sensitivity was observed for the DNLPFG with a diameter of 32 µm (period of 570 µm) coated with a sol-gel mixture of thymol blue and zinc oxide. Additionally, a small stretching force was required for the sensor to obtain higher sensitivity.

### 4.3. CO_2_ Gas Concentration Robust Sensing Experiment

**Figure 10:**
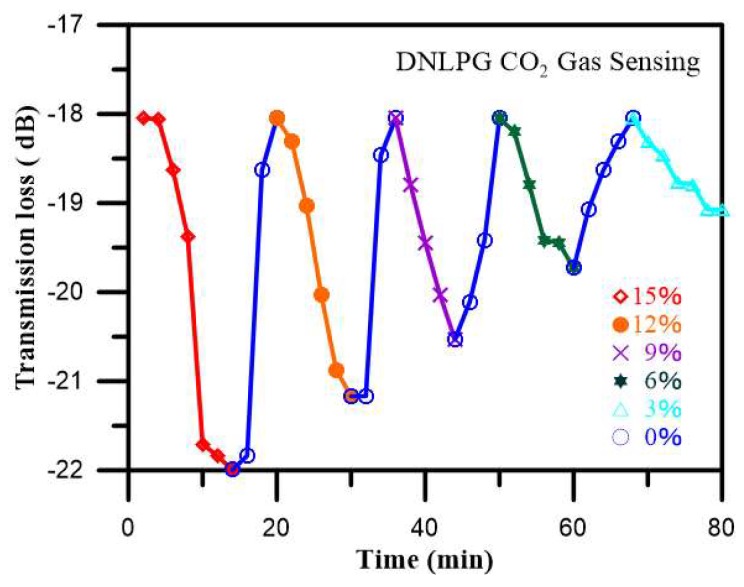
The online monitoring diagram of the 15%, 12%, 9%, 6%, and 3% CO_2_ gas concentrations over three cyclic experiments.

**Figure 11:**
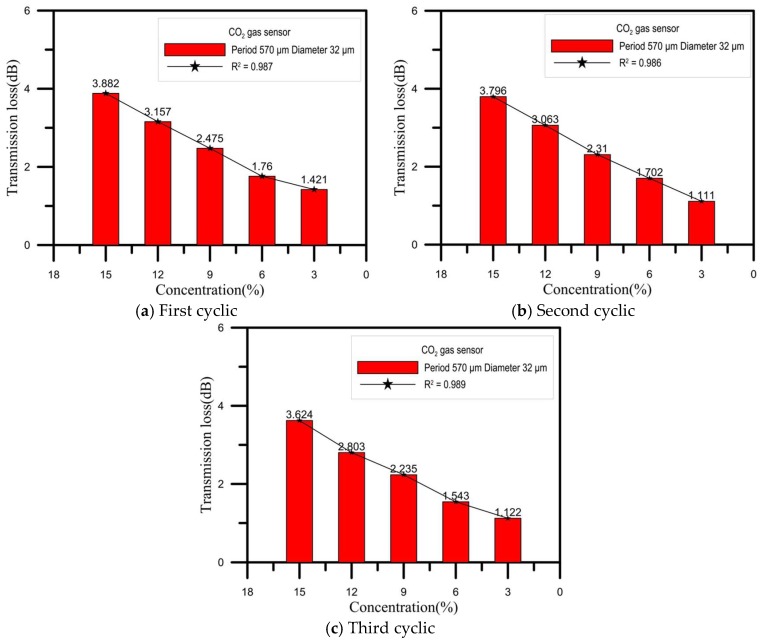
Response characteristics of sensing for the 15%, 12%, 9%, 6%, and 3% CO_2_ gas concentrations over three cyclic experiments. (**a**) First cyclic; (**b**) second cyclic; (**c**) third cyclic.

**Figure 12:**
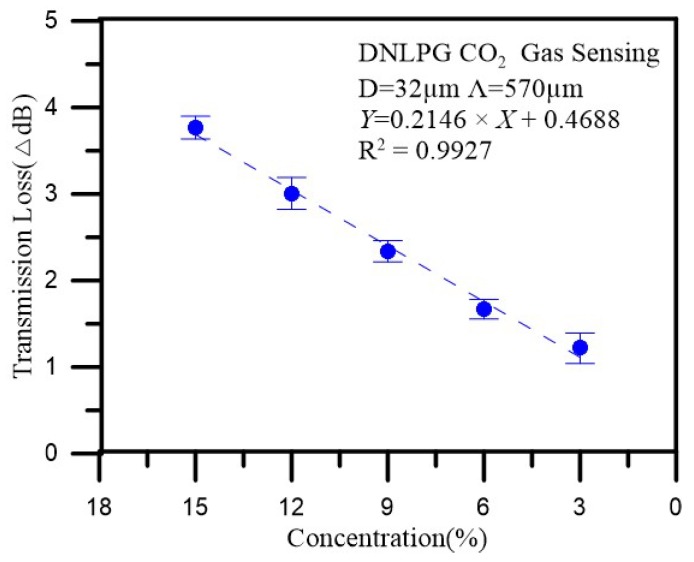
Repeatability of the CO_2_ gas sensor spectral analysis.

For this experiment, we used the 32 μm diameter, 570 μm period optic fiber coated with a sol-gel mixture of thymol blue and zinc oxide and then completely sealed in a box. An initial load of nitrogen was input into the box until the CO_2_ gas sensor spectrum was stable. Next, CO_2_ was introduced into the box at a concentration of 15% for 15 min, and the spectral changes were recorded, after which air was introduced to the box for 5 min to allow the sensor to recover and stabilize. Next, CO_2_ was introduced into the box at a concentration of 12% for 15 min, during which the spectral changes were recorded, and after which air was introduced to the box for 5 min to allow the sensor to recover and stabilize. This procedure was then repeated for CO_2_ concentrations of 9%, 6%, and 3%. Figure 10 shows the changes in loss for the 15%, 12%, 9%, 6%, and 3% CO_2_ gas concentrations sensed in three cyclic experiments. In Figure 11, we can clearly see the intensity and transmission loss variations of different concentrations of CO_2_ in the three cyclic. As indicated, we found that when the gas was detected, the coupling coefficients for the cladding and core became smaller, decreasing the transmission loss, which could be verified using Equation (2). Figure 12 shows the repeatability of the CO_2_ gas sensor spectral analysis. The mean and standard deviation changes in transmission loss for CO_2_ at concentrations of 15%, 12%, 9%, 6%, and 3% were 3.7673 dB, 3.0077 dB, 2.3400 dB, 1.6683dB, and 1.2180 dB (as shown in Table 1), respectively. As the CO_2_ concentration increased, the transmission loss increased; sensitivity was 0.2146 dB/% and linearity was 0.992.

According to the literature [17,18,19], the maximum transmission loss variation of previously reported sensors was 2.50 dB, while the associated sensitivity was approximately 0.089 dB/% (as shown in Table 2). In this study, in which we coated DNLPFG with thymol blue mixed with ZnO, the maximum transmission loss variation was 3.881 dB and the sensitivity was approximately 0.2146 dB/%. The maximum transmission loss variation and the sensitivity were thus 56% (|(3.881 − 2.50)/2.50|) and 141% (|(0.2146 − 0.089)/0.089|) higher, respectively, than those for the best sensors previously reported in the literature.

## 5. Conclusions

This study used DNLPFG gas sensors with diameters of 40, 35, and 32 μm, respectively, and coated them with a mix of thymol blue and ZnO as a sensing layer using the sol-gel method to detect 3% to 15% concentrations of CO_2_ gas. When the diameter of the fiber was 32 μm (and the period was 570 μm) and the CO_2_ gas concentration reached 15%, the results showed that the maximum loss variation reached up to 3.881 dB, while the sensitivity was 0.2146 dB/% and the linearity was 0.992. This maximum transmission loss and sensitivity were thus 56% and 141% better, respectively, than those for the best sensors previously reported in the literature. These results demonstrate that the DNLPFG CO_2_ gas sensor is quite sensitive in terms of detecting CO_2_ at room temperature.

## Figures and Tables

**Table 1:** Mean and standard deviation changes in loss for the different CO_2_ gas concentrations.

CO_2_ Concentration	Mean Loss (dB)	Standard Deviation of Loss (dB)
15%	3.7673	0.1313
12%	3.0077	0.1833
9%	2.3400	0.1227
6%	1.6683	0.1123
3%	1.2180	0.1758

**Table 2:** Comparison with different sensors used to measure CO_2_ concentrations.

Ref.	Sensing Film	Concentration	Transmission Loss Variation	Sensitivity
[17]	Thymol blue	0–100%	2.500 dB	0.0250 dB/%
[18]	PEHA	0–15%	2.490 dB	0.0727 dB/%
[19]	TEPA	0–15%	2.019 dB	0.0890 dB/%
In this study	Thymol blue ZnO	0–15%	3.881 dB	0.2146 dB/%
